# Integrative analysis unveils ECM signatures and pathways driving hepatocellular carcinoma progression: A multi‐omics approach and prognostic model development

**DOI:** 10.1111/jcmm.18230

**Published:** 2024-04-03

**Authors:** Zhen Liu, Pengfei Zhao

**Affiliations:** ^1^ Department of Radiology Shengjing Hospital of China Medical University Shenyang China

**Keywords:** drug selection, extracellular matrix, immune infiltration, immunotherapy, liver hepatocellular carcinoma, single‐cell sequencing

## Abstract

Liver hepatocellular carcinoma (LIHC) is a highly lethal form of cancer that is among the deadliest cancer types globally. In terms of cancer‐related mortality rates, liver cancer ranks among the top three, underscoring the severity of this disease. Insufficient analysis has been conducted to fully understand the potential value of the extracellular matrix (ECM) in immune infiltration and the prognostic stratification of LIHC, despite its recognised importance in the development of this disease. The scRNA‐seq data of GSE149614 was used to conduct single‐cell analysis on 10 LIHC samples. CellChat scores were calculated for seven cell populations in the descending cohort to investigate cellular communication, while PROGENy scores were calculated to determine tumour‐associated pathway scores in different cell populations. The pathway analysis using GO and KEGG revealed the enrichment of ECM‐associated genes in the pathway, highlighting the potential role of the ECM in LIHC development. By utilizing the TCGA‐LIHC cohort, an ECM‐based prognostic model for LIHC was developed using Lasso regression. Immune infiltration scores were calculated using two methods, and the performance of the ECM‐related risk score was evaluated using an independent cohort from the CheckMate study. To determine the precise expression of ECM‐associated risk genes in LIHC, we evaluated hepatocellular carcinoma cell lines using a range of assays, including Western blotting, invasion assays and Transwell assays. Using single‐cell transcriptome analysis, we annotated the spatially‐specific distribution of major immune cell types in single‐cell samples of LIHC. The main cell types identified and annotated included hepatocytes, T cells, myeloid cells, epithelial cells, fibroblasts, endothelial cells and B cells. The utilisation of cellchat and PROGENy analyses enabled the investigation and unveiling of signalling interactions, protein functionalities and the prominent influential pathways facilitated by the primary immune cell types within the LIHC. Numerous tumour pathways, including PI2K, EGFR and TGFb, demonstrated a close correlation with the involvement of ECM in LIHC. Moreover, an evaluation was conducted to assess the primary ECM‐related functional changes and biological pathway enrichment in LIHC. Differential genes associated with ECM were identified and utilised to create prognostic models. The prognostic stratification value of these models for LIHC patients was confirmed through validation in multiple databases. Furthermore, through immune infiltration analysis, it was discovered that ECM might be linked to the irregular expression and regulation of numerous immune cells. Additionally, histone acetylation was mapped against gene mutation frequencies and differential expression profiles. The prognostic stratification efficacy of the ECM prediction model constructed in the context of PD‐1 inhibitor therapy was also examined, and it exhibited strong stratification performance. Cellular experiments, including Western blotting, invasion and Transwell assays, revealed that ECM‐associated risk genes have a promoting effect on the development of LIHC. The creation of biomarkers for LIHC using ECM‐related genes unveiled substantial correlations with immune microenvironmental infiltration and functional mutations in various tumour pathways. This enlightens us to the possibility that the influence of ECM on tumours may extend beyond simply promoting the fibrotic process and the stromal composition of tumours.

## 1 INTRODUCTION

Liver hepatocellular carcinoma (LIHC) is a highly lethal form of cancer that ranks among the top three cancer types with the highest mortality rates worldwide.^1^ HCC is the primary type of liver cancer, comprising over 90% of all primary liver cancers.^2^, ^3^ At present, surgery is considered the most effective treatment option for early‐stage HCC.^4^, ^5^ Unfortunately, due to the often asymptomatic nature of HCC, less than 30% of patients are eligible for surgical treatment, and many patients cannot receive the most effective diagnosis and treatment options due to the advanced stage of the disease.^6^ Chemotherapy is typically ineffective against HCC, and treatment options for early‐stage HCC are often limited to surgical resection, liver transplantation and local excision.^7^, ^8^ Despite its limited benefits of only a few months of prolonged survival, sorafenib remains the primary systemic agent for treating advanced hepatocellular carcinoma.^9^, ^10^ Although liver transplantation can greatly enhance the survival rates of HCC patients, obstacles such as tumour recurrence, immune rejection and a shortage of available liver donors pose significant challenges.^11^, ^12^, ^13^ With the incidence of liver cancer on the rise, clinical phase I/II trials of immune checkpoint inhibitors (ICIs) are being conducted in patients with advanced liver cancer, with the potential to pave the way for immunotherapy in liver cancer following the remarkable success of immunotherapy in some patients with advanced or nearly advanced melanoma or lung cancer.^11^, ^12^, ^13^ Despite the promise of immunotherapy for LIHC, there is significant variation in treatment sensitivity among patients, which significantly impacts clinical treatment decisions. This underscores the importance of conducting in‐depth analyses to explore the association between tumour heterogeneity and treatment resistance, with the goal of improving treatment outcomes for patients with LIHC.

In recent years, there has been growing interest in the impact of the microenvironment on cancer tissue, with particular attention paid to the role of the extracellular matrix (ECM) in HCC development. The ECM has been studied in depth, with research revealing its involvement in various aspects of HCC progression, such as tumour growth, apoptosis, drug resistance, invasion, and metastasis.^14^ The ECM is a complex network of molecules with diverse biochemical properties that regulate various cellular functions, including cell growth, survival, motility and differentiation.^15^ The ECM is a dynamic structure that is constantly modified to maintain tissue homeostasis. Its components are in constant interaction with each other, and their isolation and local release of growth factors and other signalling molecules act as ligands for cell receptors, delivering signals such as cell adhesion, migration, proliferation and apoptosis.^16^, ^17^ The ECM serves as a critical regulator of cellular processes and functions and plays a crucial role in tissue development, homeostasis and repair. Furthermore, the ECM is under constant reconstruction and remodelling by cells, involving complex processes such as synthesis, degradation, reorganisation and chemical modification. These processes require strict regulation to maintain tissue homeostasis. Disruption of ECM homeostasis is a hallmark of cancer and often marks critical events in tumour progression and metastasis. Therefore, the ECM plays a dual role as both a structural support for tumour growth and an essential component of the tumour microenvironment that contributes to the development of HCC. Despite this, there is currently no prognostic model for LIHC based on the ECM, and the precise mechanisms and specific functions of the ECM in the development and immune infiltration of LIHC remain largely unexplored.

Given the significant role of ECM in the development of LIHC and the evidence that major ECM components contribute to the pathogenesis of LIHC, there is a need for further exploration of the specific mechanistic functions of ECM and the development of a prognostic model for LIHC based on ECM. We conducted an investigation into the role of ECM in LIHC development by analysing a large transcriptomics database. This allowed us to identify the key mechanisms by which ECM contributes to LIHC, including its role in promoting immune infiltration and invasive metastasis. Our findings suggest that targeting ECM may be a promising strategy for developing new therapies for LIHC and modulating immune infiltration in the tumour microenvironment. We utilised single‐cell transcriptome analysis to annotate the spatial distribution of major immune cell types in LIHC samples. By using CellChat and PROGENy analysis, we identified the signalling interactions, protein functions and key pathways that are mediated by these immune cell types in LIHC. We also evaluated the enrichment of significant functional alterations and biological pathways related to the ECM in LIHC. We screened for differentially expressed genes related to the ECM to construct prognostic models and validated their prognostic value in stratifying LIHC patients across multiple databases. Additionally, our study investigated the potential correlation between ECM and abnormal expression and regulation of various immune cells through immune infiltration analysis. Furthermore, we performed histone acetylation mapping against gene mutation frequencies and differential expression profiles and evaluated the prognostic stratification ability of the ECM prediction model in the context of PD‐1 inhibitor treatment. This approach may offer technical and intellectual support for the identification of novel targets for LIHC treatment and for addressing resistance to immunotherapy.

The current use of machine learning in studying the ECM (extracellular matrix) in liver cancer is primarily focused on several areas. Firstly, machine learning algorithms can be utilised to analyse the ECM features present in liver cancer tissue and provide more accurate predictions of disease progression and prognosis. An example of the application of machine learning in the study of ECM in liver cancer is the use of classification algorithms to differentiate between different types of liver cancer based on the ECM components present in the tissue. This approach has the potential to improve the accuracy of diagnosis and treatment planning for liver cancer. Machine learning algorithms have been utilised to analyse the ECM composition and pathways in liver cancer treatment in order to support the development of more effective treatment plans. For instance, researchers have employed machine learning algorithms to analyse the ECM components in liver cancer tissue and used this information to predict the treatment response of liver cancer patients, which in turn could help optimise treatment plans. Therefore, the current study aims to utilise machine learning methods to investigate the crucial role of ECM in HCC.

## 2 MATERIALS AND METHODS

### 2.1 Data acquisition

The patient data used in this study were obtained from The Cancer Genome Atlas (TCGA, https://portal.gdc.cancer.gov/) and Gene Expression Omnibus (GEO, https://www.ncbi.nlm.nih.gov/geo/) DataSets. These publicly available datasets provide comprehensive gene expression data and clinical information, including high‐throughput sequencing data of LIHC tumour and adjacent non‐tumour tissues, as well as clinical profiles. The TCGA database was searched for public gene expression data and complete clinical annotations, which included bulk RNA‐seq from 368 patients with liver cancer in the TCGA‐LIHC cohort, as well as bulk RNA‐seq from 20 patients with liver cancer from GSE14520 and scRNA‐seq data from 10 single liver cancer cells from GSE149614. The transcriptomic data and clinical profiles of TCGA‐LIHC cohort were extracted and processed using Perl (v5.30.0) for subsequent analysis. We transformed bulk sequencing data into Transcripts Per Million (TPM) format for subsequent analysis. Gene annotation was performed on TCGA‐LIHC and GSE14520 based on platform information, and the intersection of gene symbols was taken. We ensured the consistency of feature scales between the two datasets using the z‐score normalisation method. The Combat function from the “sva” package was employed to remove batch effects in the two bulk data sets. Patient samples with any data abnormalities were excluded. Quality control and data cleaning for GSE149614 were conducted using the “Seurat” package. Our criteria were set as follows: percent.mt <10, nCount_RNA >1000, nFeature_RNA between 100 and 5000.

Each patient's clinical data in the study included various information such as age, gender, ethnicity, pathological histological classification, tumour stage, recurrence, survival time and clinical outcome. For cohorts with more comprehensive clinical data, genomic sequencing data and transcriptome sequencing data (e.g., LncRNA, microRNA) were also included in the analysis. The studies were selected based on specific inclusion and exclusion criteria. The inclusion criteria were: (1) primary LIHC confirmed by post‐operative or intra‐operative pathology; (2) patients with at least 30 days of clinical follow‐up after diagnosis and (3) complete follow‐up clinical data including genomic sequencing data. The exclusion criteria were: (1) secondary liver cancer or post‐liver transplantation; (2) presence of multiple liver tumours or other types of liver tumours and (3) poor outcome after liver intervention or immunotherapy, etc.

### 2.2 Cellular annotation and ECM‐related spatial distribution profiling of LIHC single‐cell samples

Conventional second‐generation sequencing (NGS) analyses the genome of a group of cells, including cell cultures, tissues, organs, or entire organisms, providing an “average genome” of a cell population. In contrast, single‐cell sequencing determines the genome of individual cells within a cell population. These conventional techniques are commonly referred to as bulk sequencing, in contrast to single‐cell technologies. At present, single‐cell sequencing techniques can be employed to measure the genome (scDNA‐seq), DNA methylome, or transcriptome (scRNA‐seq) of individual cells within a population. These methods have been utilized to discover novel mutations in cancer cells, investigate the dynamic epigenomic changes that occur during embryonic development and evaluate the heterogeneous expression patterns of specific genes within an otherwise seemingly homogeneous population of cells. Thus, the aim of our study was to investigate the predominant distribution of ECM and the potential distribution of gene expression in LIHC. To achieve this, we focused on genes associated with the ECM pathway (obtained from the MSigDB database, https://www.gsea‐msigdb.org/gsea/msigdb/index.jsp), including ARHGAP5, DIAPH1, FYN, GSN, HRAS, ITGB1, MAP2K1, MAPK1, MAPK3, MYL2, PFN1, PIK3CA, PIK3CG, PIK3R1, RAF1, RHOA, ROCK1, SHC1 and TLN1 (Table S1). Furthermore, we analysed scRNA‐seq data from 10 LIHC samples from GSE149614, which were divided into 7 cell populations. High‐throughput single‐cell histology data is characterised by a large volume of data and a vast number of cells that can be analysed simultaneously. To handle such data, a new dimensionality‐reducing manifold learning technique called Uniform Manifold Approximation and Projection (UMAP) has been developed. UMAP is based on the principles of Riemannian geometry and algebraic topology and is a powerful algorithm for reducing high‐dimensional data into a lower‐dimensional representation while preserving the essential structure of the data. UMAP is a powerful dimensionality reduction algorithm that can produce high‐quality visualisations of large‐scale single‐cell data. Compared to t‐SNE, another popular dimensionality reduction algorithm, UMAP can preserve more global structure, has better runtime performance, and is more scalable. Additionally, UMAP can be used as a general dimensional reduction technique for machine learning without computational restrictions on the number of embedding dimensions. In this study, UMAP was used for clustering cells based on filtered principal components, which allowed for cell classification and visualisation. The collected data was processed and downscaled to construct seven cell subpopulations. The spatial distribution of single cells from 10 LIHC patients was visualised to gain insight into the expression of cell types in different LIHC tumours. ECM‐specific markers from previous studies were used to label the identified cell types and their spatial distribution. A hierarchical bar chart was used to compare and analyse the percentage and distribution of cell types in different LIHC patients.

### 2.3 Signalling communication and biological pathway enrichment analysis of ECM's major gene annotation for LIHC single‐cell sequencing

After preprocessing and downsampling of single‐cell scRNA‐seq data from GSE149614, single‐cell analysis was performed to identify relevant genes and functional pathways. The pathway analysis was carried out using PROGENy, which predicts the activity of signalling pathways, and other pathway signatures were introduced to reflect the activation of Notch, MYC and PIM. This analysis helped distinguish between oncogenic and tumour suppressor pathways and identify the main factors influencing patient survival. To understand the main ECM‐mediated pathways in LIHC, we computed PROGENy scores on single‐cell data from LIHC to investigate the impact of ECM on the development of LIHC with respect to key tumour‐acting pathways. Following this, we computed CellChat scores to analyse multiple cell populations obtained by downscaling, revealing the cellular communication functions and modes of action of different cell types in LIHC under the influence of ECM, particularly those identified in single‐cell transcriptome sequencing networks. The method employs single‐cell expression profiles of ligands, receptors and cofactors (including heterodimeric molecular complexes) to determine the mutual strength of cell–cell communication, avoiding the limitation of considering only one ligand‐receptor gene pair and neglecting the involvement of multi‐subunit receptors. The ligand‐receptor pairs that exhibit significant interactions are detected through a probability‐based ligand‐receptor interaction analysis and a perturbation test. The resulting intercellular communication networks are then generated by aggregating the number or strength of significant ligand‐receptor interactions between different cell types. Furthermore, we also calculated GSVA enrichment pathway scores for three distinct cell types using 50 Hallmark datasets. To compare two groups, ECM signature‐high and ECM signature‐low, we utilized the FindAllMarkers function in the Seurat package to screen for differentially expressed genes (DEGs) with adjusted *p*‐values < 0.05 and absolute values of logFC >0.585.

GO (Gene Ontology) is a standardised system for classifying gene functions internationally. It consists of a set of constantly updated standard vocabularies that describe the characteristics of genes and gene products in an organism in a comprehensive manner. The fundamental building blocks of GO are terms, which represent attributes associated with genes and gene products. The KEGG (Kyoto Encyclopedia of Genes and Genomes) database is a comprehensive database that integrates genomic and functional information, including metabolic pathway databases, hierarchical classification databases, gene databases, genome databases and more. The KEGG pathway database is the most commonly used public database for metabolic pathways. It is used to systematically analyse gene functions and link them to pathways. In this study, we utilised the KEGG pathway database to investigate the tumour biology and identify enrichment pathways related to ECM‐related genes. To evaluate the significance of a GO term in the prospective genes of interest, we compared the proportion of that term in these genes with the proportion of the term in all background genes. This analysis was performed using the “clusterProfiler” and “enrichplot” R packages, which enabled implementation of GSEA analysis. The results were displayed graphically, showing the top five GO functions and KEGG pathways ranked by their scores. The visualisation facilitated easy interpretation of the findings.

### 2.4 Biological construction and clinical validation of prognostic risk models for LIHC with ECM‐related genes

Cox univariate analysis was performed on the differentially expressed genes using the “survival” data package, from which hazard ratio (HR) values and *p*‐values were obtained. The intersection genes with *p*‐value less than 0.01 from the two survival analyses were extracted using R software, and the differentially expressed intersection genes were used as individual variables for Cox multifactor analysis. The stepwise feature selection method was utilized to identify the most informative gene variables from the differential intersection genes, resulting in gene variable X, regression coefficient β and HR values. Next, a prognostic model was constructed by selecting prognostically significant genes from the 22 genes based on Least absolute shrinkage and selection operator (Lasso) regression using TCGA‐LIHC cohort. The Lasso algorithm was implemented using the “Glmnet” package, with our iterative parameter set to 10,000. We employed cross‐validation to select the λ that minimised the average error (lambda.min). At this point, the corresponding genes and coefficients formed the optimal model genes and coefficients. The model risk score was calculated by multiplying the expression levels of each gene by its corresponding coefficient and then summing these values. The formula is as follows:

$$\text{Riskscore}=\sum_{i=1}^{n}\left({\text{Gene}}_{i}\;\text{expression value}*{\text{Gene}}_{i}\;\text{coefficient}\right)$$

The forest plots were drawn using the “survminer” package to visualize the HR results. The “predict” function was used to calculate the survival risk score (SRS) for each tissue based on the RS formula, and the tissues were classified into high‐risk and low‐risk groups using the median SRS of all tissues. The correlation between SRS and prognosis was verified using the Kaplan–Meier method for survival analysis, and the accuracy of the RS prognostic model was evaluated by plotting receiver operating characteristic (ROC) curves using the “survivalROC” data package. The prognostic model was validated externally using 20 patients with liver cancer from cohort GSE14520. We generated receiver operating characteristic (ROC) curves to assess the diagnostic performance of the signature within the test cohort. An area under the curve (AUC) greater than 0.7 was considered indicative of a robust diagnostic performance.

### 2.5 ECM risk model for association analysis of related genes with LIHC immune infiltration and evaluation of treatment sensitivity

To investigate the relationship between immune infiltration and the progression of hepatocellular liver cancer, this study employed the ssGSEA method to calculate different immune cell scores and immune‐related function scores for all hepatocellular carcinoma samples from the training set. Subsequently, differential analyses were performed to compare the high and low‐risk groups based on sample groupings constructed from the prognostic model. A *p*‐value less than 0.05 was considered statistically significant. The “limma” R package was utilised for analysing the differences between high‐ and low‐risk groups, and the results were visualised using the “ggpubr” R package through box plots of immune cell infiltration and immune function. In addition, the “limma” R package was applied directly for performing differential analysis of immune checkpoint‐related gene expression between high‐ and low‐ risk groups, with a significance threshold of *p* < 0.05. The “ggpubr” R package was used for creating visual maps of the results. To ensure accurate predictions, we also used the Xcell algorithm to calculate immune infiltration scores, which were then visualised using boxplots, heatmaps and scatterplots. Tissues are complex environments consisting of different types and subtypes of cells, each with their own unique transcriptome. Therefore, in bulk transcriptome analysis, the expression of genes specific to each cell type is weighted by the proportion of that cell type in a given sample. The process of deconvoluting gene expression profiles enables the reconstruction of the cellular makeup of tissues. Through the use of xcell, a robust computational approach, gene expression profiles can be transformed into detailed fractions representing 64 different immune and stromal cell types across various samples. By analysing differences in cell type composition across individuals, cellular targets of diseases can be identified, leading to the exploration of novel therapeutic strategies. Moreover, adjusting for these variables enables the detection of genuine gene expression differences and enhances the comprehension of downstream analyses. Building upon this, we investigated and elaborated on the influence of immune infiltration and tumour immune interactions in an ECM‐related prognostic model of LIHC. In line with the methodology employed in the analysis of single‐cell sequencing data to compute ECM signature, we independently computed the ECM‐related gene signatures for the training and validation cohorts. Subsequently, these signatures were categorised into ECM‐high and ECM‐low groups based on their respective median values. To assess the correlation between the ECM signature obtained through the “xCELL” algorithm and immune cell scores for each patient, Pearson correlation analysis was employed. The resulting differences between the two groups were visualised using a heatmap. *p* < 0.05 was considered statistically significant. Additionally, we performed a mutational analysis of the TCGA‐LIHC cohort and used the “maftools” R package (version 2.12.0) to visualise the mutation data. We evaluated the performance of the risk score using an independent cohort from the CheckMate study, which comprised of patients with metastatic uroepithelial cancer treated with the PD‐1 inhibitor nivolumab.

### 2.6 Cell culture and transfection

The hepatocellular carcinoma HepG2 and SNU‐387O2 cell lines were obtained from the ATCC cell bank. For aerobic culture, the cells were maintained in a constant temperature cell incubator (37°C, 5% CO_2_) and cultured in high‐glucose Dulbecco's modified eagle medium (DMEM) supplemented with 10% foetal bovine serum and 1% penicillin–streptomycin. Regular digestion, fluid exchange and passage were performed.

In this study, we investigated the role of SPP1 in hepatocellular carcinoma by downregulating its expression in two cell lines through various phenotypic experiments. We divided each cell line into three groups: the control group, the si‐control group and the si‐SPP1 group. We commissioned a biotech company (Sangon Corporation, China) to design and produce siRNA for SPP1 and a negative control group. The siRNA was dissolved based on the manufacturer's recommendations. We seeded two cell lines at a density of 4 × 10^5^ cells per well in a 6‐well plate, and each group was supplemented with 2.5 mL of culture medium. Twenty‐four hours before transfection, all groups were switched to serum‐free medium for cell starvation. The siRNA, dissolved in 10 μL, was mixed with 125 μL of Opti‐MEM medium (Thermo, USA), and the transfection reagent Lipofectamine 3000 (Thermo, USA) was dissolved in an appropriate amount according to the instructions in 125 μL of Opti‐MEM medium. The siRNA and transfection reagent mixture was incubated in the dark at room temperature for 20 min, centrifuged at 2000 rpm for 1 min and then evenly added to the corresponding groups. After 4 h, each group was replaced with complete culture medium, and subsequent experiments were conducted 48 h post‐transfection.

### 2.7 Cell total protein extraction and western blot

The culture medium from each cell group was discarded, and after washing three times with phosphate‐buffered saline (PBS), a protein lysis mixture (Beyotime, China, RIPA:PMSF = 100:1) was added. After thorough cell lysis by pipetting, the preliminary protein lysate was transferred to corresponding Eppendorf tubes, followed by additional sonication under the following parameters: amplitude 25%, 1 s each time, three times per group. All Eppendorf tubes were placed on ice for further protein lysis for 30 min, vigorously shaking every 10 min. The lysate was then centrifuged at 12,000 rpm for 20 min at 4°C, and the supernatant was collected for protein concentration determination using a spectrophotometer.

Based on the protein concentration, an appropriate amount of loading buffer was added, and the mixture was heated in a metal bath for 5 min. After cooling, 20 μg per lane of each sample was loaded into precast 10% SDS‐PAGE gels (Epizyme, China). Electrophoresis was carried out at 135 V for 70 min, and the proteins were transferred onto a 0.45 μM PVDF membrane (Millipore, USA). After blocking the PVDF membrane with a fast blocking solution (Beyotime, China) for 15 min, the membrane was cut and then incubated with the corresponding primary antibodies at 4°C for 12 h. After washing with Tris‐buffered saline with Tween (TBST), the membrane was incubated with secondary antibodies for 1 h. Protein bands were visualised using an enhanced chemiluminescence (ECL) substrate (Beyotime, China) on a machine. All antibodies were obtained from Proteintech Group (China).

### 2.8 CCK8 assay

After transfection for 24 h, HepG2 and SNU‐387O2 cell lines were separately transferred to a 96‐well plate at a density of 5000 cells per well, with PBS covering the perimeter to prevent evaporation. Upon observing cell adhesion 24 h later, CCK‐8 reagent (Biosharp, China) was mixed with DMEM complete medium according to the manufacturer's instructions, and 200 μL of the mixture was added to each well to avoid bubble formation. The 96‐well plate was wrapped in aluminium foil to shield it from light and placed in an incubator. After a 3‐h incubation period, absorbance at 450 nm for each well was measured using a microplate reader. The procedure was repeated at 24, 48, 72 and 96‐h time points. Three replicate wells were set up for each group. This analysis aimed to ascertain whether there were statistically significant differences among the groups.

### 2.9 Colony formation assay

The cells from each group in the six‐well plate were digested using trypsin (Biosharp, China) and resuspended in DMEM‐complete medium. The cells were then seeded in a new six‐well plate at a density of 1000 cells per well, with 3 mL of DMEM complete medium added to each well. After 14 days of cultivation, the culture medium was removed, and each well was fixed with 1 mL of 4% paraformaldehyde for 30 min, followed by two washes with PBS. Subsequently, 1 mL of crystal violet staining solution was added to each well and allowed to stain for 10 min. After two washes with PBS, images were captured and cell colony counting was performed.

### 2.10 Transwell assay

We employed the Transwell assay to investigate differences in the invasive capabilities of cells in each group. Initially, the ECM gel (Corning, USA) was diluted at a 1:8 ratio, and 30 μL of the diluted gel was added to each well of the Transwell chamber (Thermo, USA) and allowed to dry in a cell culture incubator. In a 24‐well plate, 600 μL of DMEM complete medium was added to each well. After 48 h of transfection, cells from each group were digested from the six‐well plate, resuspended in DMEM culture medium without FBS, and 30,000 cells were added to each chamber, with the chamber liquid volume brought up to 200 μL. The chamber was placed in a 24‐well plate and incubated in a cell culture incubator for 24 h. Subsequently, the liquid in the chamber was removed, and the cells were fixed with 4% paraformaldehyde for 30 min, followed by two washes with PBS. Staining was performed with 0.1% crystal violet for 20 min. After three washes with PBS and thorough drying, images were captured under a microscope, and cell counting was conducted.

### 2.11 Statistical analysis

All analyses in this study were conducted using R software (V4.1.3). Unless otherwise specified, the “ggplot2” package served as the primary tool for visualization. For statistical analysis of intergroup differences in Western blot, colony formation assay and Transwell assay, *t*‐tests were employed. Statistical analysis for the CCK8 assay was performed using two‐way ANOVA. A significance threshold of *p* < 0.05 was considered statistically significant (*p < 0.05; **p < 0.01; ***p < 0.001; ****p < 0.0001).

## 3 RESULTS

### 3.1 Cellular annotation and ECM‐related spatial distribution profiles depicted for LIHC single‐cell samples

**FIGURE 1:**
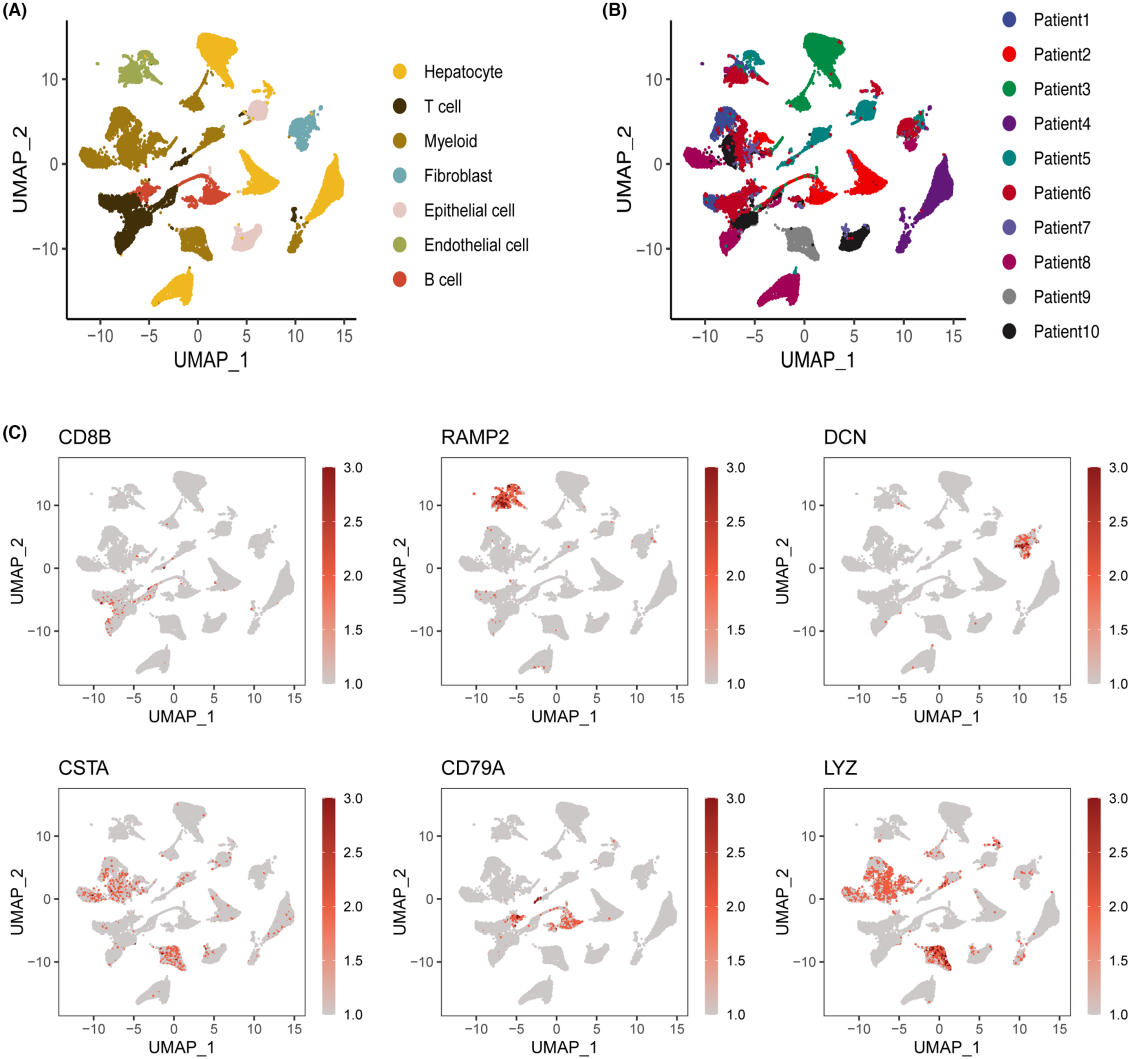
Cell annotation and ECM‐related spatial distribution profiles depicted for LIHC single‐cell samples. (A) The scRNA‐seq data from GSE149614 were subjected to UMAP dimensionality reduction, followed by the annotation of seven cellular subtypes (hepatocyte, T cell, myeloid, fibroblast, epithelial cell, endothelial cell and B cell). (B) The distribution of cell samples from 10 patients in GSE149614 on the UMAP plot is depicted. (C) The expression profiles of ECM‐related genes (CD8B, RAMP2, DCN, CSTA, CD79A and LYZ) across various cellular subtypes are illustrated.

Single‐cell analysis was conducted on 10 LIHC samples using scRNA‐seq data from GSE149614. UMAP was utilised to successfully cluster cells and perform downstream analysis. The obtained single‐cell sequencing data were annotated based on their taxonomic content differences and colour‐coded according to their spatial distribution. Seven main cell groups were identified and annotated through clustering (Figure 1A): hepatocyte, T cell, myeloid, fibroblast, epithelial cell, endothelial cell and B cell. These cell groups were spatially distributed and exhibited differences in content based on cell type, as illustrated by the distribution of images. Considerable variation was observed in the spatial distribution and content of different cell types, with B cells and endothelial cells exhibiting a high degree of spatial dependence. Subsequently, the cellular enrichment and spatial annotation of the 10 LIHC patients were specifically annotated (Figure 1B). Each patient's spatial distribution was paired with their respective cell types. Samples from patients 1, 2, 4 and 8 were mainly located in the hepatocyte distribution area, while patients 3 and 7 showed a higher expression of epithelial cells in their spatial annotation. The spatial distribution of other patients' samples also showed some variation. Additionally, to demonstrate the spatial distribution and role dependence of ECM‐associated genes in LIHC single‐cell annotation, we specifically annotated the cellular distribution of LIHC single‐cell samples using ECM‐related gene markers (Figure 1C).

The markers used for the cellular distribution of LIHC single‐cell samples were RAMP2, DCN, CSTA, CD8B, CD79A and LYZ. Among them, RAMP2 was found to be mainly distributed in the endothelial cell region, while DCN was co‐expressed with fibroblasts. CSTA was mainly secreted by myeloid cells, and CD8B was expressed at relatively low levels. CD79A was identified as the main marker of B cells as in previous studies, while LYZ was mainly co‐distributed with myeloid cells.

**FIGURE 2:**
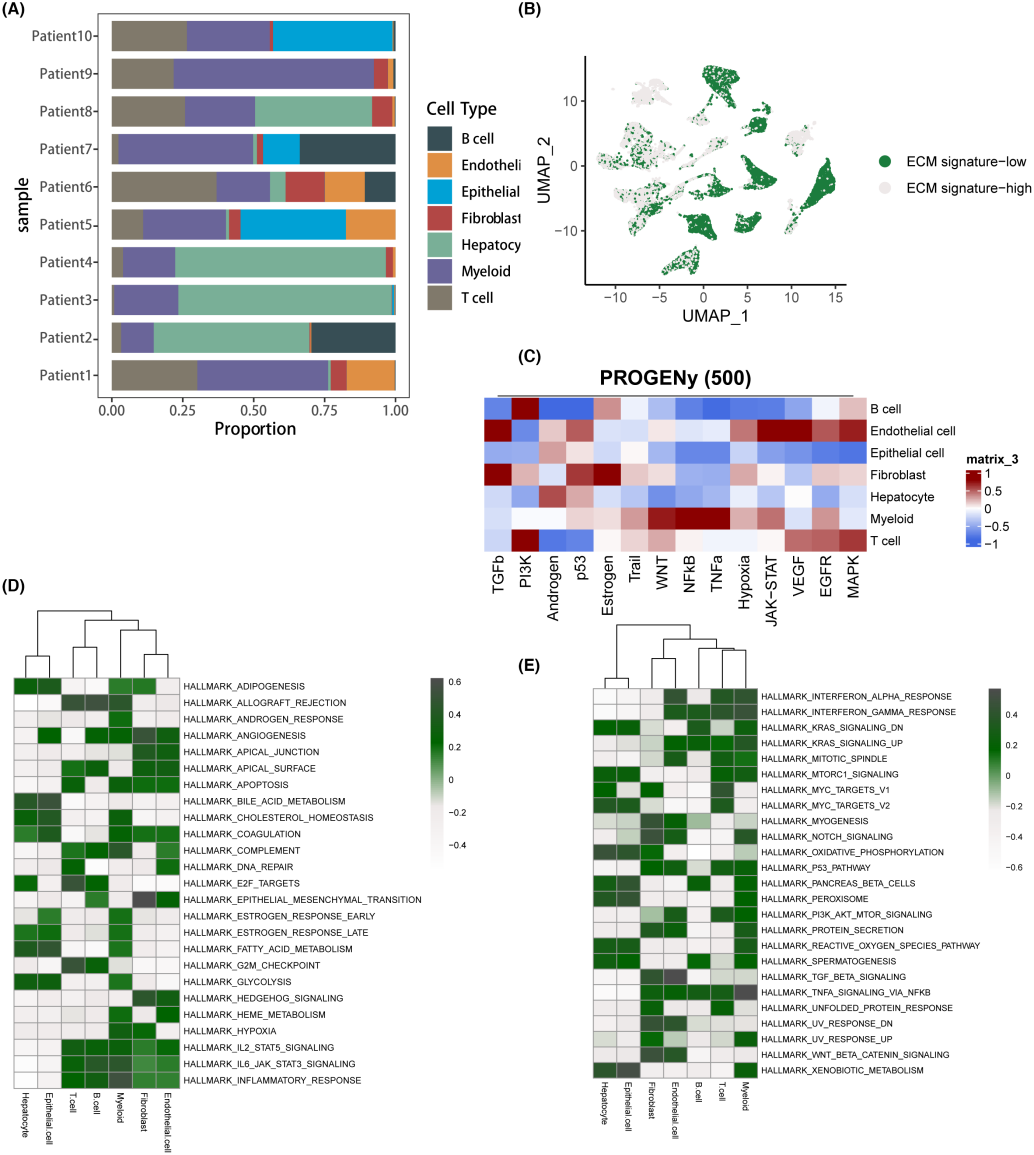
Cluster distribution and tumour functional pathway network of LIHC single‐cell samples. (A) Histogram depicting the distribution of cellular subtypes among the 10 patients in GSE149614. (B) Visualization of the spatial distribution of single cells categorized into ECM signature‐high and ECM signature‐low groups based on gene set enrichment scoring. (C) Heatmap illustrating the tumour‐related pathway scores for each cellular subtype, calculated using the PROGENy algorithm. (D) Enrichment analysis of HALLMARK pathways using ECM GSVA in the signature‐high group, with green indicating high expression. (E) Enrichment analysis of HALLMARK pathways using GSVA in the CM signature‐low group, with green indicating high expression.

A hierarchical histogram (Figure 2A) was used to analyse the cell type distribution of the 10 LIHC patients. The histogram showed that the majority of patients had single‐cell samples composed mainly of T cells, myeloid and epithelial. Patient 1 had higher expression of endothelial, while patients 2, 3 and 4 had samples composed mainly of hepatocytes. To measure the activity of the ECM pathway of each cell line, we used the Seruat package with the AddModuleScore function. The scores were then divided into two groups based on the median score value: ECM signature‐high and ECM signature‐low (Figure 2B). The findings indicated that the regions with high expression of ECM were predominantly located in stromal components such as endothelial cells, myeloid cells, fibroblasts and T cells. This suggests that ECM components play a major role in the proliferative and regenerative components of the liver, which is consistent with our previous understanding of the role of ECM in chronic liver disease.

### 3.2 Signalling communication and biological pathway enrichment analysis of key gene annotations of LIHC

**FIGURE 3:**
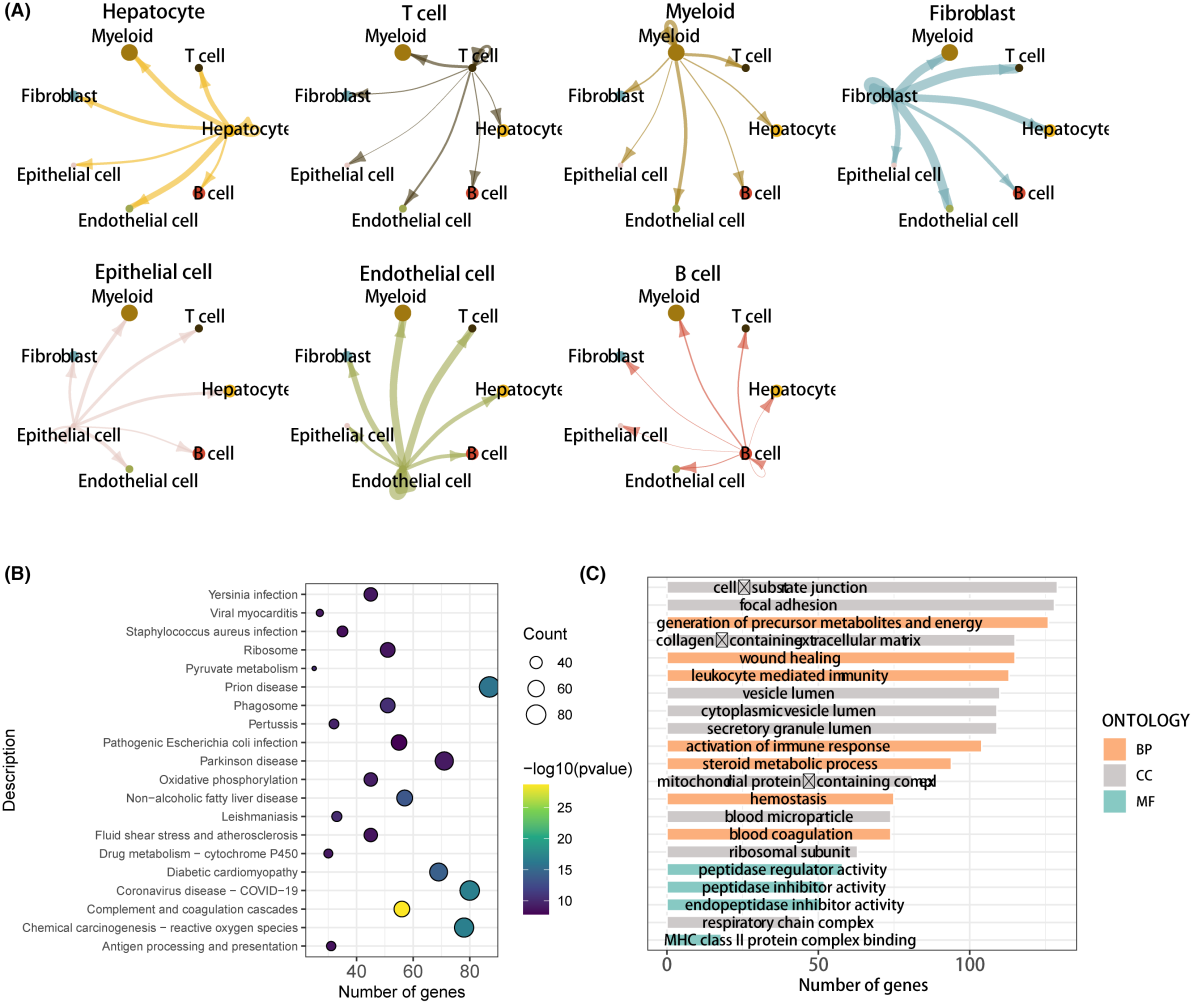
Signalling communication and biological pathway enrichment analysis of major gene annotations for LIHC. (A) Analysis of cellular communication between various cellular subtypes and other subtypes. (B) KEGG enrichment bubble plot depicting differentially expressed ECM genes in scRNA‐seq data. (C) Summary of pathways obtained from GO analysis.

Following our initial examination of the distribution of immune cell types in LIHC single‐cell samples, we aimed to annotate the tumour pathways and biological functions related to ECM expression. To accomplish this, we used the PROGENy score to uncover the expression correlation of various cell types with the primary tumour pathways (Figure 2C). The PROGENy score revealed that epithelial cells had low relevance to tumour signalling pathways. The oncogenic role of fibroblasts in LIHC was dominated by TGFb, p53 and oestrogen, while the tumour‐associated pathway for T cells was PI3K, and myeloid cells were enriched in NFKb and TNFa. We calculated GSVA enrichment pathway scores for both the ECM signature‐high and ECM signature‐low groups. In the ECM signature‐high group (Figure 2D), ADIPOGENESIS, ALLOGRAFT_REJECTION, ANGIOGENESIS, SURFACE, COAGULATION, IL2_STAT5_SIGNALLING, IL6_JAK_STAT3_SIGNALLING and INFLAMMATORY_RESPONSE were significantly enriched for expression. In the ECM signature‐low group (Figure 2D), INTERFERON_ALPHA_RESPONSE, INTERFERON_GAMMA_RESPONSE, KRAS_SIGNALLING_DN, KRAS_SIGNALLING_UP and MITOTIC_SPINDLE were the major expression‐enriched genes. This also underscores a noteworthy association between ECM expression and the enrichment of tumour functional pathways. To investigate the immune microenvironment of LIHC and identify potential therapeutic targets for the disease, we utilised the scRNA‐seq data to quantitatively infer and analyse intercellular communication networks. We predicted intercellular communication by integrating the interactions between gene expression, signalling ligands, receptors and their cofactors. Thus, Figure 3A presents a comparison and analysis of the signalling interactions and communication among the major cell types annotated in LIHC. The results demonstrate that hepatocyte cells primarily signal to myeloid cells, and T cells also communicate more with myeloid. Myeloid cells predominantly signal to T cells and B cells, while fibroblasts show significant cellular communication with myeloid, B cells, T cells and endothelial cells. The types of cells that received signals from epithelial cells were more evenly distributed. Regarding endothelial cells, the main signal receivers were myeloid, T cells and fibroblasts, and B cells were the main signal recipients with limited function as signal emitters.

Based on our previous findings, we aimed to investigate the main functional pathways and biological mechanisms that contribute to the role of the ECM in LIHC. To achieve this, we performed KEGG and GO analyses. KEGG analysis (Figure 3B) revealed that the differential genes associated with ECM were mainly enriched in Yersinia infection and viral myocarditis. The GO analysis (Figure 3C) revealed an enrichment of biological pathways for the differential genes associated with ECM. The analysis revealed significant enrichment in various cellular components such as cell‐substrate junction, focal adhesion, collagen‐containing extracellular matrix, vesicle lumen, blood microparticle and ribosomal subunit. Enrichment of biological pathways included the generation of precursor metabolites and energy, wound healing, activation of immune response in the lumen, steroid metabolic process and blood coagulation. Molecular functional enrichment results focused on activities such as peptidase regulator, peptidase inhibitor, endopeptidase inhibitor and MHC class ll protein complex binding.

### 3.3 Biological construction and clinical validation of prognostic risk models for LIHC with ECM‐associated genes

**FIGURE 4:**
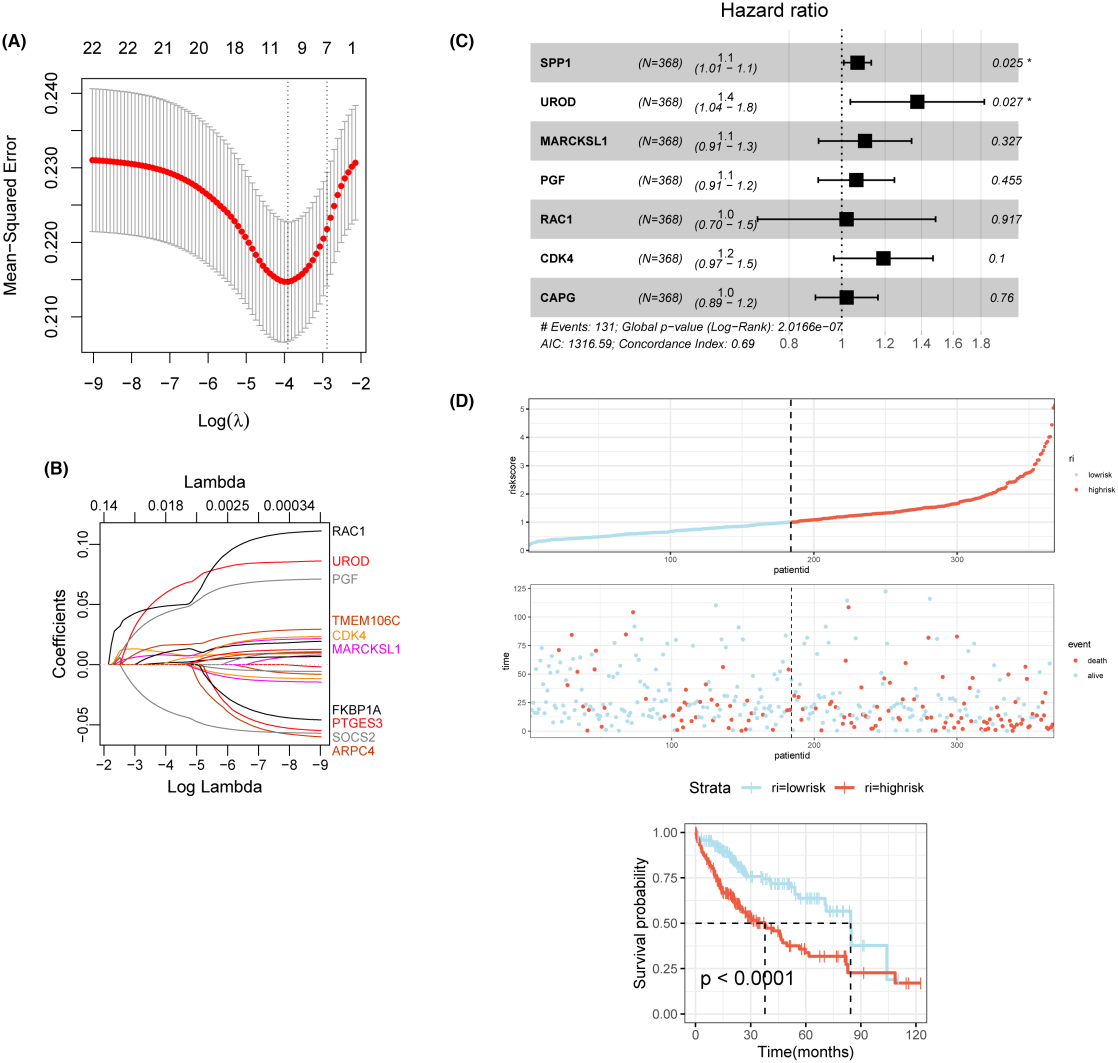
Biological construction and clinical functional validation of prognostic risk models for LIHC with ECM‐associated genes. (A) Cross‐validation curve for LASSO regression analysis. (B) Coefficient path plot for LASSO regression analysis. (C) Forest plot of univariate COX regression analysis for model genes. (D) Risk accumulation factor plot and survival curves for two risk groups.

We conducted further analysis to explore the clinical application and prognostic value of ECM in LIHC based on its important relationship with tumour infiltration and progression, as identified in the previous study. We analysed the functional impact and pathway association of ECM‐related genes on LIHC, searched for key risk genes among the differentially expressed ECM genes and developed a prognostic model. Using the TCGA‐LIHC cohort, we selected seven prognostic ECM genes from 22 genes using Lasso regression and constructed a prognostic model. The selected ECM genes with prognostic significance were SPP1, UROD, MARCKSL1, PGF, RAC1, CDK4 and CAPG. The feature screening and optimal cut‐off screening process of LASSO regression are shown in Figure 4A. The study used the median risk score to classify patients into low‐risk and high‐risk groups based on the calculated risk score using the following formula: risk score = (SPP1 * (−0.06648715)) + (UROD * 0.31881952) + (MARCKSL1 * 0.09811402) + (PGF * 0.06122678) + (RAC1 * 0.02001780) + (CDK4 * 0.17574060) + (CAPG * 0.02062824). To determine the prognostic significance of the ECM genes included in the model, univariate COX regression was performed and the forest plot in Figure 4C displays the prognostic weights of the major risk genes, with SPP1 and UROD identified as the main risk genes. The prognostic value of the ECM risk score in TCGA‐LIHC was evaluated using survival curves, scatter plots depicting survival distribution and Kaplan–Meier curves. The analysis indicated that high‐risk group patients, with higher ECM expression, had poorer survival rates (*p* < 0.01) compared to those in the low‐risk group, who exhibited significantly longer survival times. This finding was confirmed by survival scatter plots and line plots shown in Figure 4A.

In Figure S1A, we validated the prognostic stratification ability of the constructed model for LIHC patients using an independent validation cohort of 20 liver cancer patients from GSE14520, by applying K‐M survival curves, scatter plots and line plots (*p* < 0.05). The results of ROC curve showed that signature in the testing cohort had good test efficiency (AUC = 0.705). Moreover, we analysed the expression correlation of several ECM‐related genes incorporated into the model using a correlation heat map (Figure S1B). The results showed a significant co‐expression profile between RAC1 and several other genes, as well as between MARCKSL1 and multiple ECM risk genes, indicating that most ECM‐related risk genes are independent predictors of prognosis in LIHC. In Figure S1C, the distribution of ECM risk genes in the low‐ and high‐risk groups for ECM was demonstrated using box plots. The expression of all seven risk genes was significantly higher in the high‐risk group of LIHC than in the low‐risk group.

Furthermore, in order to improve the clinical applicability of the ECM risk model and to meet the clinical decision‐making needs of multifactoriality, we performed multifactorial COX regression analysis to investigate the association between clinical indicators and risk scores and developed a comprehensive scoring model. Valid clinical indicators, including gender, age and Riskscore composition, were included in the final model, which was screened by COX analysis. The ECM risk prognostic model for LIHC is presented in Figure S1D. Additionally, we analysed the chromosomal distribution of the ECM risk genes (Figure S1E). The location of each gene was as follows: MARCKSL1 and UROD were located on chromosome 1, CAPG on chromosome 2, SPP1 on chromosome 4, RAC1 on chromosome 7 and CDK4 and PGF on chromosomes 13 and 14, respectively.

### 3.4 Analysis of the tumour biology of ECM‐mediated LIHC immune infiltration

**FIGURE 5:**
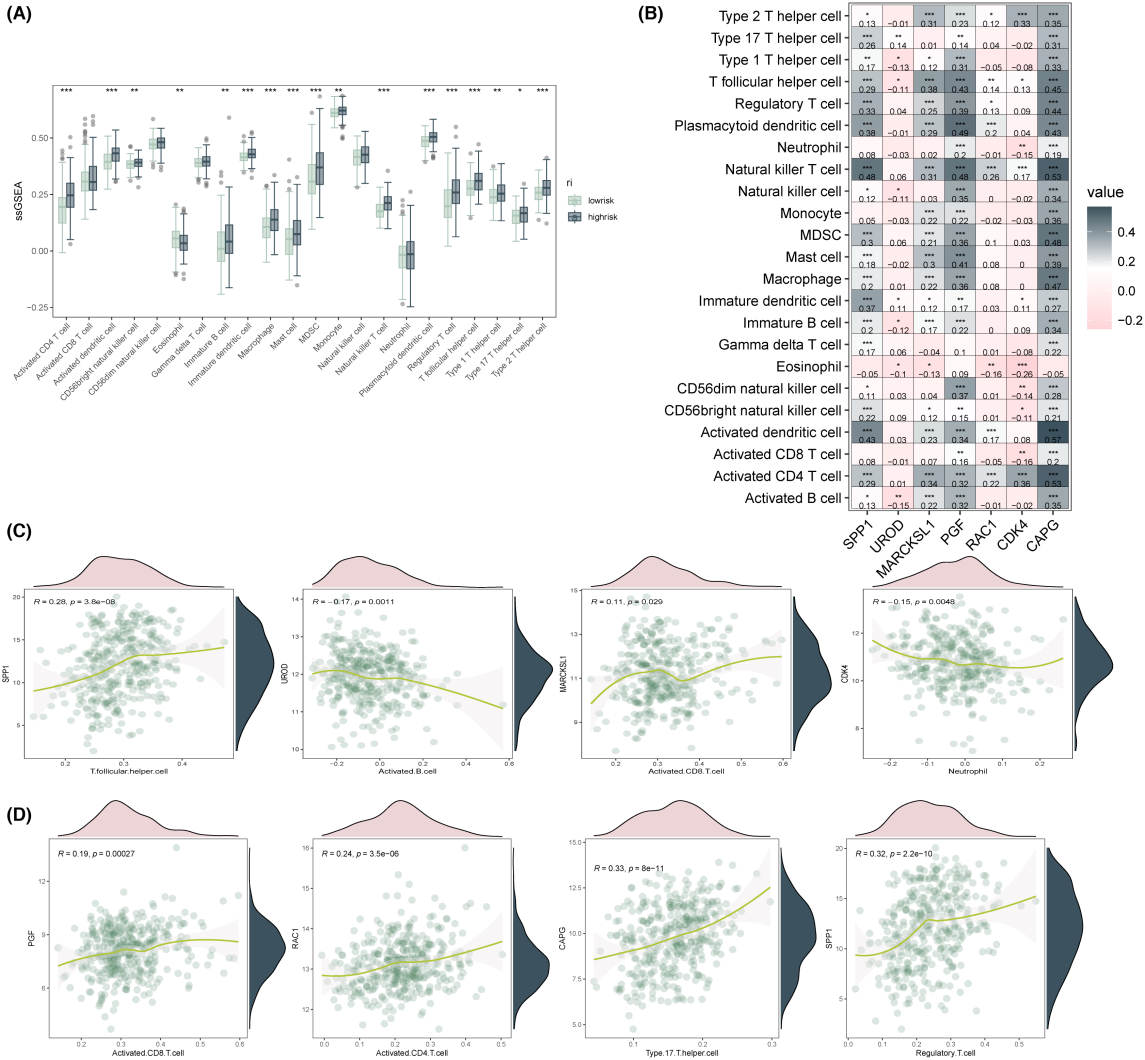
LIHC immune infiltration analysis of ECM‐associated genes. (A) Visualisation of 22 immune cell infiltration scores for two risk groups, calculated based on the ssGSEA algorithm, presented through box plots. (B) Heatmap depicting the correlation between model genes and the scores of 22 immune cell infiltrations. (C) Scatter plots illustrating the correlation between the expression levels of certain model genes and the scores of specific immune cells. (D) Scatter plots illustrating the correlation between the expression levels of certain model genes and the scores of specific T cells.

Based on the impact of the pooled ECM‐associated genes on LIHC tumour pathways and biological functions, it is suggested that the regulatory role of ECM risk genes in LIHC is associated with the immune infiltration of tumours. This association may enable us to better identify the immune infiltration status and regulatory features of LIHC. Hence, we conducted an analysis to investigate the impact of immune infiltration‐related risk genes on LIHC and their association with the regulation of immune cell expression and immune infiltration. We evaluated immune infiltration scores using two methods: ssGSEA and the xCell algorithm, and visualised the results using box line plots, heat maps and scatter plots. The ssGSEA method was used to calculate the enrichment scores for individual samples and gene set pairs to quantify the level of immune infiltration. Based on the analysis of immune infiltration results from the transcriptional data, we observed that the expression of activated and purified forms of CD3+ T cells, CD8+ T cells, cytotoxic lymphocytes, NK cells, B lymphocytes, cells derived from monocytes (monocyte lineage), myeloid dendritic cells, neutrophils and endothelial and fibroblasts was significantly higher in the high‐risk group of ECM compared to the low‐risk group. However, there was no significant difference in the distribution of endothelial cells between the two groups. Linear correlation scatter plots were used to calculate and depict the correlation between the expression of ECM risk score and immune cell expression, as shown in Figure 5C. In addition, we also calculated the correlation between the distribution of various tumour immune cells and found that there was a significant and specific correlation between the intra‐tumour distribution of multiple immune cells, as shown in Figure 5B. The analysis revealed several significant correlations between ECM risk genes and immune cell expression in LIHC. Specifically, UROD was found to have a significant negative correlation with activated B cell expression, while SPP1 was positively correlated with Th cell expression. MARCKSL1 showed a negative correlation with activated CD8 T cell expression, and CDK4 was negatively correlated with neutrophil expression. Other significant correlations between the expression of ECM risk genes and immune cell populations are shown in Figure 5D, including a positive correlation between SPP1 and conventional T cell expression, a positive correlation between PGF and activated CD8 T cell expression, a positive correlation between RAC1 and activated CD4 T cell expression, and a positive correlation between CAPG and Type17th cell expression. Figure S2A,B depict the correlation between ECM expression and tumour immunity in LIHC using the XCell algorithm. ECM signature calculations for both the training and validation cohorts showed that patients in the ECM‐HIGH group generally had significantly increased levels of immune cell infiltration. Figure S2C,D display the mutation data of the TCGA‐LIHC cohort using the R package “maftools” (version 2.12.0). The mutation patterns of the ECM in the low‐risk and high‐risk groups were significantly different. The high‐risk group was primarily associated with TP53 mutations, whereas the low‐risk group was primarily associated with CTNNB1 mutations. Figure S2E illustrates the correlation between the ECM risk score and the expression of multiple immune cells and immune checkpoint genes. The histogram results demonstrate a strong association between the ECM and tumour immunity in LIHC. We evaluated the performance of the riskscore in an independent cohort from the CheckMate study, which included patients with metastatic uroepithelial cancer treated with the PD‐1 inhibitor nivolumab. The results (Figure S2F) indicate that patients in the high‐risk group exhibited better responsiveness to PD‐1 inhibitors, suggesting that the hub genes could serve as potential targets for immunotherapy.

### 3.5 Experimental validation of ECM risk genes in hepatocellular carcinoma cell lines

**FIGURE 6:**
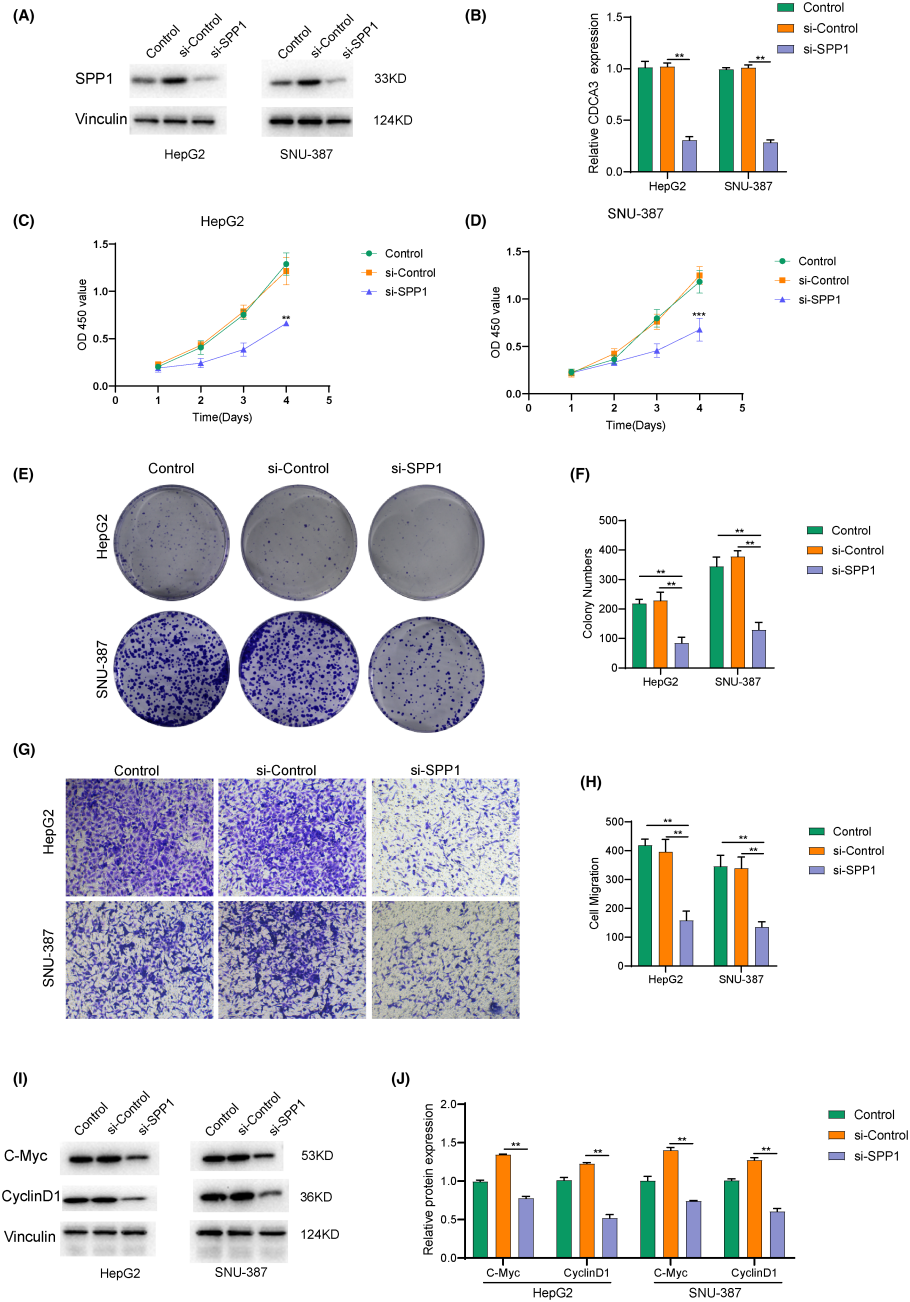
Validation of cellular level ECM‐related genes producing effects on hepatocellular carcinoma. (A) Verification of the knockdown efficiency of SPP1 in HepG2 and SNU‐387 cell lines through Western blot. (B) Quantitative analysis of the results of SPP1 knockdown efficiency in HepG2 and SNU‐387 cell lines through Western blot. (C) Time‐dependent changes in absorbance of three groups of HepG2 cells in CCK8 experiments. (D) Time‐dependent changes in absorbance of three groups of SNU‐387 cells in CCK8 experiments. (E) Photographs of colony formation in two cell lines with three groups of cells in plate colony formation experiments. (F) Statistical analysis of the number of colonies formed in each group in plate colony formation experiments. (G) Photographs depicting the invasive effects of three groups of cells from two cell lines in Transwell experiments. (H) Statistical analysis of the number of invasive cells in each group in Transwell experiments. (I) Verification of the protein expression differences of C‐Myc and CyclinD1 in different groups in HepG2 and SNU‐387 cell lines through Western blot. (J) Quantitative analysis of the protein expression differences of C‐Myc and CyclinD1.

Building on this, we sought to confirm the direct impact of ECM‐related genes on the development and invasive metastasis of hepatocellular carcinoma through cellular experiments. Accordingly, we conducted relevant experiments, beginning with knockdown of ECM‐related genes in two hepatocellular carcinoma cell lines. In Figure 4C, unfavourable prognostic impacts were identified through univariate Cox regression analysis for SPP1 and UROD (HR > 1). Notably, the statistical significance was more pronounced for SPP1 (*p* = 0.025). For this purpose, we chose the SPP1 gene from the ECM risk model. As demonstrated by WB experiments (Figure 6A), the expression of SPP1 was significantly reduced in the Si‐SPP1 group as compared to the control group, Si‐control. The bar graph in Figure 6B further demonstrated that SPP1 expression was significantly lower in the Si‐SPP1 group compared to the control and Si‐Control groups. After obtaining cell lines with SPP1 knockdown, we investigated the effect of SPP1 knockdown, an ECM risk gene, on hepatocellular carcinoma cell lines using the CCK8 assay. The results indicated that as the culture time increased, both HepG2 (Figure 6C) and SNU‐387 (Figure 6D) cell lines exhibited significantly lower cell survival in the Si‐SPP1 group than in the control and Si‐Control groups, suggesting that SPP1 might promote the growth of hepatocellular carcinoma cells in some way, consistent with the bioinformatic analysis in our previous study. To further investigate the impact of SPP1 on the invasive metastasis of hepatocellular carcinoma, we conducted transwell assays (Figure 6E,G). We compared the effects of SPP1 knockdown on tumour colony formation and invasive metastasis in two hepatocellular carcinoma cell lines. The results demonstrated (Figure 6F,H) that knockdown of SPP1 significantly inhibited colony formation as well as invasion and metastasis in both hepatocellular carcinoma cell lines. These findings suggest that ECM‐related genes, such as SPP1, play a crucial role in promoting both the growth and invasive metastasis of hepatocellular carcinoma. These results motivate us to explore the underlying mechanisms behind the effects of SPP1 on hepatocellular carcinoma. Our experiments revealed that the knockdown of SPP1 in both hepatocellular carcinoma cell lines led to a significant reduction in the expression of C‐Myc and Cyclin‐D1 (Figure 6I), indicating that SPP1 may promote tumour development through the myc signalling pathway. The histogram (Figure 6J) further confirms the statistically significant difference between the knockdown group and the control group. These findings provide strong evidence for the direct association between ECM and hepatocellular carcinoma, highlighting the role of SPP1 in promoting tumour development through the myc signalling pathway.

## 4 DISCUSSION

In the past few years, sequencing technologies have advanced significantly, particularly with the advent of single‐cell transcriptome sequencing. This technology has allowed researchers to better classify cell types in tumour tissue and measure gene expression levels in individual cells, which has greatly advanced the field of tumour immunology. Recently, single‐cell transcriptome sequencing has been applied to the study of the immune status of hepatocellular carcinoma, leading to some important findings. Previous studies have highlighted the enrichment of certain cell subsets, such as depleted CD8T cells and Treg cells, which are preferentially found in HCC. The upregulation of the gene layilin in activated CD8T cells and Treg cells has been shown to suppress CD8T cell function.^18^, ^19^ In our study, we aimed to investigate the role of ECM in regulating the tumour microenvironment and immune infiltration in LIHC patients, as well as the specific mechanisms and pathways involved. To achieve this, we utilised single‐cell sequencing to analyse the distribution of ECM‐related genes in immune cells within the LIHC tumour microenvironment. Simultaneously, we developed a prognostic risk model to achieve efficient prognostic stratification of LIHC patients and investigated the correlation between TEX, immune infiltration and differential expression of immune cells. Furthermore, we experimentally validated the promotive effect of ECM‐related genes on tumour development through cellular‐level experiments, which serves as a basis for further exploring the underlying mechanisms of how ECM‐related genes influence the altered tumour microenvironment in liver cancer.

The disruption of ECM homeostasis is widely recognised as a hallmark of cancer, signifying critical events in tumour progression and metastasis. Consequently, ECM serves not only as a structural scaffold for tumour growth but also as a crucial element of the tumour microenvironment, intricately involved in the process of HCC development. Current studies on the ECM have delved into the genetic level, concentrating on the precise control of ECM remodelling. FN, which is an important component of the ECM, has been found to play crucial roles in various cellular processes such as cell adhesion, migration, growth and differentiation. FN binds to the extracellular fibulin‐1/calmodulin/senescence key protein antigen 1 complex and relies on cell surface molecules, such as syndecan‐4 and integrin α5β1, to inhibit ERK1/2 signalling and cell migration.^20^ FN1 triggers polymorphic expression of the interferon‐α type 1 receptor (IFNαR1) promoter, indicating that the PI3K/Akt signalling pathway is significantly disrupted in HCC cells. Furthermore, extracellular FN1 can activate this signalling pathway.^21^ Studies have demonstrated that a reduction in the protein levels of FN1 and integrin β1 can disrupt the PI3K pathway.^22^ Xu et al.^23^ identified FN1 as a target of the transcription factor CP2 (TFCP2) in HCC cells and a crucial mediator of HCC metastasis. The TFCP2 binding site motif was found in the promoter of FN1. Inhibiting the FN1 transcriptional‐translational program can prevent the increased invasiveness of HCC cells induced by overexpressed TFCP2. Integrins α5β6 and α9β1 have been found to be involved in the migration of cancer cell aggregates on FN‐rich substrates. It has been shown that integrin α5β6‐driven cell migration occurs independently of underlying transforming growth factor β (TGF‐β) activation and Smad‐dependent signalling in tumour cells.^24^ Collagen is a crucial protein component of the ECM that provides tensile strength and restricts tissue expansion. Various types of collagen are present in HCC tissue, with type I collagen alpha 1 (COL1A1) being the most abundant.^25^ COL1A1 has been shown to confer enhanced oncogenicity and a survival advantage to HCC cells. Silencing of COL1A1 expression using siRNA (siCOL1A1) resulted in the attenuation of Slug‐dependent epithelial‐mesenchymal transition (EMT) and HCC stemness gene markers, including SOX2, OCT4 and CD133. Consequently, siCOL1A1 inhibited the proliferative clonality, motility, invasiveness and tumour sphere formation in HCC cells. Therefore, the intricate connection between ECM and hepatocellular carcinoma development has been demonstrated by various studies. In our investigation, we confirmed the potential influence of ECM on liver tumour immune infiltration, prognostic regulation and multiple tumour biological pathways. These findings are expected to serve as a basis for the development and exploration of innovative therapies for liver cancer.

Currently, there is limited literature on the relationship and mechanistic insights of ECM with C‐Myc and Cyclin‐D in liver hepatocellular carcinoma (LIHC). In our wet experiments, the knockdown of ECM‐related gene SPP1 resulted in a significant decrease in the expression levels of C‐Myc and Cyclin‐D. This suggests that the expression levels of extracellular matrix (ECM)‐related genes can influence the expression of genes regulating the cell cycle and promoting cell proliferation within the cell. Genes within the ECM may exert their effects on the biological behaviour of tumour cells through signalling across tumour cell membranes. The trajectory of tumour cell development not only depends on its intrinsic gene expression levels but is also influenced by other cells within the ECM. The underlying mechanisms of these interactions warrant further exploration. Research by Gao Liu indicates an association between the tumour microenvironment of liver cancer and factors involved in the ECM with the anti‐tumour effect of ROR‐α‐1. Overexpression of ROR‐α‐1 significantly inhibits the proliferation, migrationand invasion capabilities of HCC (hepatocellular carcinoma) cells and downregulates the protein levels of c‐Myc and Cyclin D1, suggesting a tumour‐suppressive role for ROR‐α‐1 in liver cancer cells (PMID: 32058275). Given their findings, similar functional relationships may exist in SPP1 or other ECM‐related genes, necessitating further in‐depth investigation. The innovation of this study is that the relative expression relationship between ECM‐related genes SPP1 and C‐Myc and Cyclin‐D was verified by cytological experiments in vitro. It provides a theoretical basis for the subsequent mechanism research.

Currently, researchers have explored the impact of ECM‐related genes on the prognosis of Liver Hepatocellular Carcinoma (LIHC). Guozhi Wu et al. identified ECM‐related lncRNAs based on public datasets and constructed a prognostic model consisting of 2 lncRNAs using LASSO. Subsequently, they validated the expression differences of lncRNAs in liver cancer cell lines and normal immortalized liver cell lines through RT‐qPCR experiments. However, their model lacked external validation to assess its accuracy and the potential for overfitting (PMID: 36195857). In contrast, our study employed a combined approach of training and validation cohorts to enhance the reliability of the model. Additionally, we confirmed the role of model genes in LIHC through gene knockdown and multiple phenotypic experiments. Hui Tang et al. clustered patients based on ECM features using a public dataset and subsequently constructed a model using the LASSO algorithm. However, their model lacked external validation to assess its accuracy and did not undergo wet lab experimental validation (PMID: 35402515). In comparison, our research methodology is more accurate and rigorous, resulting in more reliable conclusions and higher practical utility.

This study presents several limitations that warrant attention in future research. Firstly, the genetic data pertaining to ECM are incomplete, and validation from diverse platform databases is essential to enhance the reliability of the findings. Secondly, additional exploration of the relationship between genomic instability and immunotherapy is crucial, given that this study exclusively concentrated on the computational framework for somatic mutation determination. Thirdly, although the distribution of ECM in immune cells within tumours and paracancerous regions was examined, the sample size of the GEO dataset utilised in this study was constrained. It is imperative to comprehend the distribution of genes associated with extracellular mechanisms in other tissues closely linked to immune cells in liver cancer, such as blood, lymph nodes and thoracic and ascitic fluid. Lastly, further functional studies conducted by experimental biologists are imperative to unravel the specific regulatory mechanisms of the identified genes. Currently, there are numerous studies on the construction of similar models, but few have been successfully applied in frontline clinical settings. The limited sample size has been a critical factor questioning the robustness of these models. Going forward, we are committed to advancing multicenter, large‐sample studies to enhance the robustness of our model, improve its practicality and contribute new insights and theoretical foundations to the improvement of treatment for LIHC.

Our study delved into the relationship between the immune system and ECM function in LIHC single‐cell samples, as well as the major cytological tumour pathways involved. By constructing LIHC biomarkers based on ECM‐related genes, we discovered significant correlations with immune infiltration in the tumour microenvironment and functional mutations in multiple tumour pathways. This suggests that the role of the ECM in tumours may extend beyond its role in promoting fibrosis and shaping the stromal composition of tumours. This information may help in the development of novel immunotherapeutic targets for LIHC and in understanding tumour physiology. In addition, this study may aid in the development of clinical therapeutic options for LIHC and the investigation of alterations in tumour microenvironment infiltration. However, there are still limitations to be addressed, such as incomplete genetic data related to ECM and the need for further experimental research to understand the specific regulatory mechanisms involved in these interactions.

## AUTHOR CONTRIBUTIONS

**Zhen Liu:** Data curation (lead); formal analysis (lead); writing – original draft (lead); writing – review and editing (supporting). **Pengfei Zhao:** Conceptualization (lead); data curation (equal); formal analysis (equal); writing – review and editing (lead).

## CONFLICT OF INTEREST STATEMENT

Not applicable.

## Supporting information


Figures S1–S2.

Table S1.

## Data Availability

All datasets generated for this study are included in the article material, including TCGA‐LIHC, GSE14520 and GSE149614.
