# Supplementary material for: Integrative analysis unveils ECM signatures and pathways driving hepatocellular carcinoma progression: A multi‐omics approach and prognostic model development
Source: J Cell Mol Med. 2024 Apr 3;28(8):e18230. doi: 10.1111/jcmm.18230 (PMC10989547; doi:10.1111/jcmm.18230)
Supplement: Supplementary file 1 — Figures S1–S2. [file JCMM-28-e18230-s001.docx]

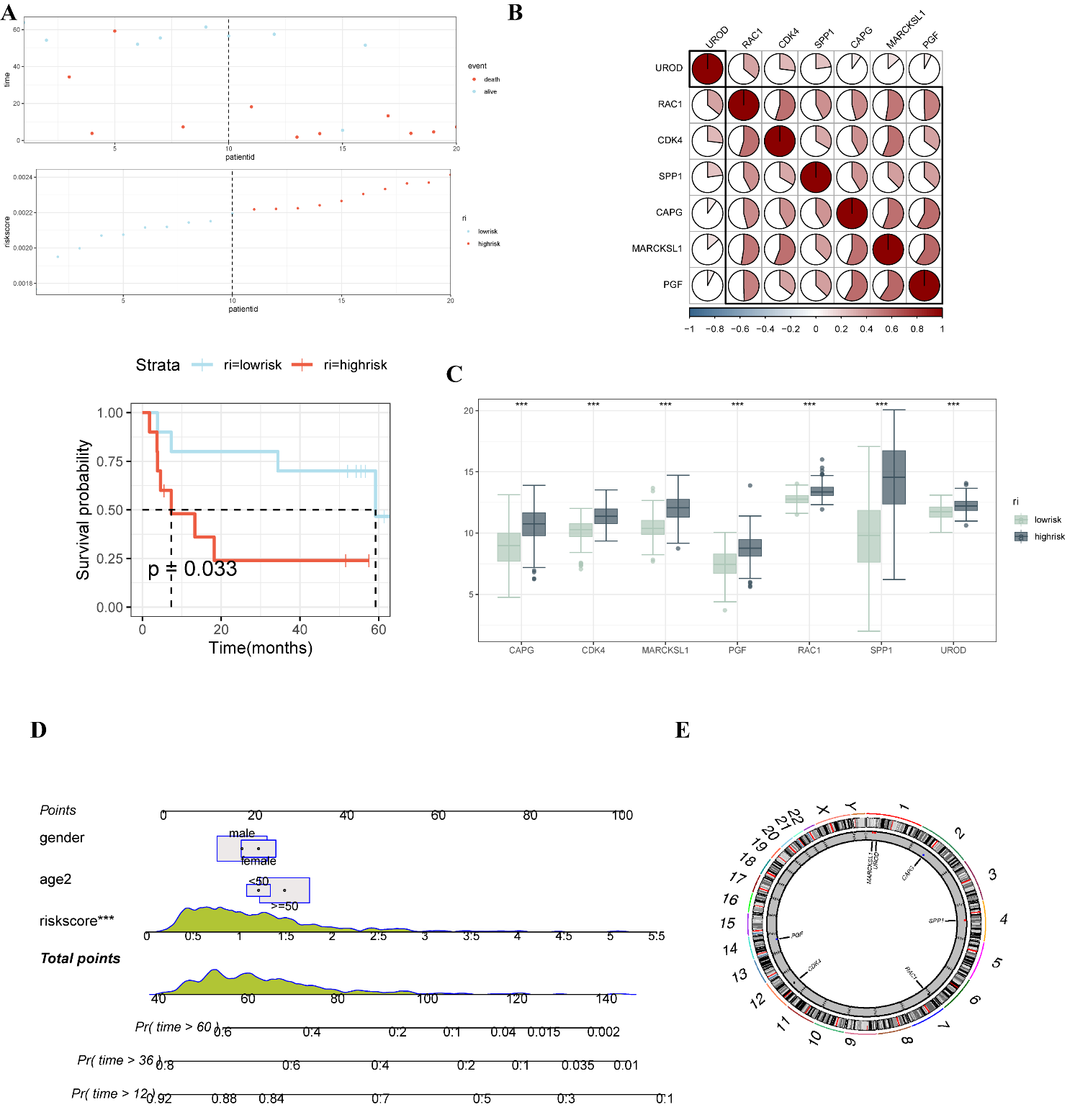


**Figure S1.** Further construction and chromosome distribution analysis of the ECM-associated genetic prognostic risk model. A. Calculation of the model using external validation cohort GSE14520 and generation of risk accumulation factor plot and survival curves for two risk groups. B. Heatmap illustrating the correlation of model gene expression in the external validation cohort. C. Box plots depicting the expression differences of model genes between two risk groups in the external validation cohort. D. Construction of a nomogram prognostic model incorporating gender, age, and Risk score in the external validation cohort. E. Circos plot representing the genomic locations of model genes on chromosomes.


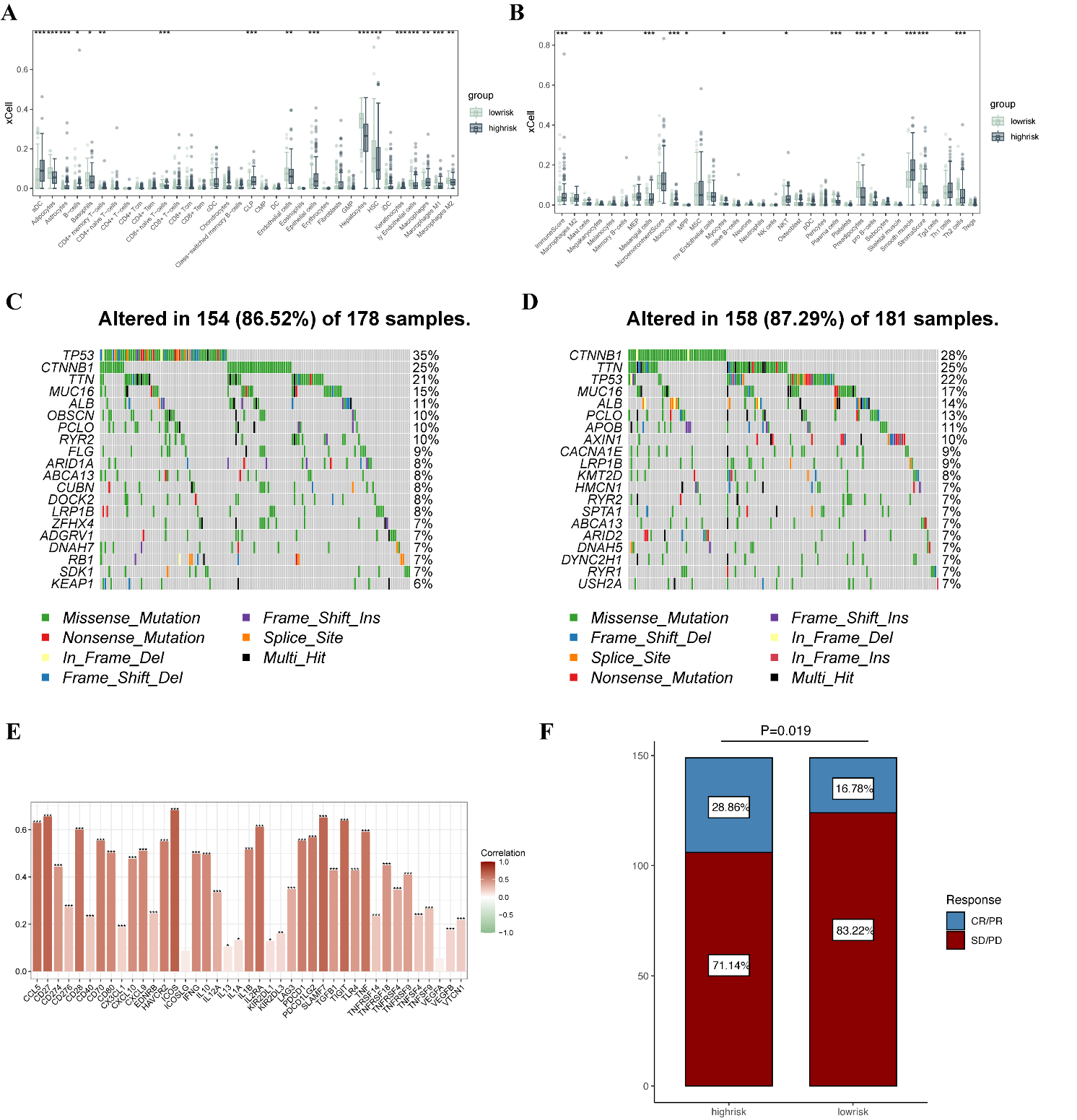


**Figure S2.** Pathway analysis and treatment sensitivity assessment of ECM-mediated LIHC immune infiltration by XCell method. A. We conducted an analysis of the differential scores of 34 immune cell types between two risk groups using the "xCell" algorithm, and visualized the results through box plots. B. Using the "xCell" algorithm, we further analyzed the differential scores of an additional 34 immune cell types between two risk groups and visualized the outcomes using box plots. C. Genetic mutation analysis of patients in the low-risk group was performed, and the results were visualized in a waterfall plot. D. Genetic mutation analysis of patients in the high-risk group was conducted, and the findings were visualized in a waterfall plot. E. We carried out a correlation analysis between ECM scores and the expression of 41 immune checkpoint genes, presenting the results through a bar chart. F. Leveraging data from the CheckMate study, we analyzed the differences in the effectiveness of immunotherapy between the high-risk and low-risk groups.
